# Discovery and structural characterization of chicoric acid as a SARS-CoV-2 nucleocapsid protein ligand and RNA binding disruptor

**DOI:** 10.1038/s41598-022-22576-4

**Published:** 2022-11-02

**Authors:** Gustavo Fernando Mercaldi, Eduardo Henrique Salviano Bezerra, Fernanda Aparecida Heleno Batista, Celisa Caldana Costa Tonoli, Adriana Santos Soprano, Jacqueline Farinha Shimizu, Alice Nagai, Jaqueline Cristina da Silva, Helder Veras Ribeiro Filho, Jéssica do Nascimento Faria, Marcos Guilherme da Cunha, Ana Carolina Mattos Zeri, Andrey Fabricio Ziem Nascimento, José Luiz Proenca-Modena, Marcio Chaim Bajgelman, Silvana Aparecida Rocco, Paulo Sérgio Lopes-de-Oliveira, Artur Torres Cordeiro, Marjorie Bruder, Rafael Elias Marques, Mauricio Luis Sforça, Kleber Gomes Franchini, Celso Eduardo Benedetti, Ana Carolina Migliorini Figueira, Daniela Barretto Barbosa Trivella

**Affiliations:** 1 Brazilian Biosciences National Laboratory (LNBio), Brazilian Centre for Research in Energy and Materials (CNPEM), Campinas, SP 13083-100 Brazil; 2 Brazilian Synchrotron Light Source (LNLS), Brazilian Centre for Research in Energy and Materials (CNPEM), Campinas, SP 13083-100 Brazil; 3 Laboratory of Emerging Viruses (LEVE), Department of Genetics, Evolution, Microbiology and Immunology, Institute of Biology, University of Campinas (UNICAMP), Campinas, SP Brazil; 4 Hub of Global Health (HGH), University of Campinas (UNICAMP), Campinas, Brazil

**Keywords:** Chemical biology, Drug screening, X-ray crystallography

## Abstract

The nucleocapsid (N) protein plays critical roles in coronavirus genome transcription and packaging, representing a key target for the development of novel antivirals, and for which structural information on ligand binding is scarce. We used a novel fluorescence polarization assay to identify small molecules that disrupt the binding of the N protein to a target RNA derived from the SARS-CoV-2 genome packaging signal. Several phenolic compounds, including L-chicoric acid (CA), were identified as high-affinity N-protein ligands. The binding of CA to the N protein was confirmed by isothermal titration calorimetry, ^1^H-STD and ^15^N-HSQC NMR, and by the crystal structure of CA bound to the N protein C-terminal domain (CTD), further revealing a new modulatory site in the SARS-CoV-2 N protein. Moreover, CA reduced SARS-CoV-2 replication in cell cultures. These data thus open venues for the development of new antivirals targeting the N protein, an essential and yet underexplored coronavirus target.

## Introduction

The historic COVID-19 pandemic, caused by severe acute respiratory syndrome coronavirus 2 (SARS-CoV-2), has caused millions of deaths worldwide and affected the world’s economy in an unprecedented way^1,2^. Despite the enormous efforts of the scientific and medical community to find drugs to fight the disease since its emergence in late 2019, only three small molecule drugs are currently authorized for clinical use. Remdesivir^3^ and Molnupiravir^4^ are nucleoside analogues with limited efficacy^5^, whereas Nirmatrelvir^6^, a peptide inhibitor of the 3CL protease, developed by Pfizer, is used in combination with Ritonavir and sold under the brand name Paxlovid™. Although these are promising drugs, kidney and liver toxicity and the potential incompatibility of the Paxlovid combination with other drugs may limit their use to the broad population^7^. Moreover, the continued emergence of new, more contagious SARS-CoV-2 variants challenges the efficacy of vaccines currently in use^8^. Therefore, the development of new and specific drugs capable of treating SARS-CoV-2 infections is still an urgent medical need.

To accelerate the discovery of novel anti-SARS-CoV-2 drug candidates, as well as the repurposing of existing drugs, several high-throughput screening (HTS) campaigns have been performed in the last two years. Such assays have explored the biological activities of several SARS-CoV-2 proteins, including the main 3C-like (3C-L) and papain-like (PL-Pro) proteases, the RNA polymerase and the spike (S) and envelope (E) structural proteins^9–14^. In addition, numerous cell-based screening assays with SARS-CoV-2 have also been recently reported^12,15–18^. Unlike the S and E proteins, however, the structural nucleocapsid (N) protein, the most abundantly expressed SARS-CoV-2 protein in infected cells, has not been fully exploited as a target for drug development against SARS-CoV-2^19^.

The N protein is a phosphoprotein that plays fundamental roles in the virus life cycle, including transcription initiation and packaging of the viral genomic RNA^20,21^. This protein has a domain architecture comprising two structured RNA-binding modules, the N-terminal (NTD) and C-terminal (CTD) domains, connected by an intrinsically disordered serine and arginine-rich (SR) linker. The NTD and CTD are also flanked by flexible ends^22–24^. The NTD is implicated in the binding and melting of regulatory elements required for transcription initiation^23,25,26^, whereas the CTD drives protein dimerization and is thought to contribute to the binding of the RNA packaging signal (PS) sequence during virus particle assembly^27,28^. Phosphorylation of the SR linker by host cell kinases, on the other hand, has been shown to influence the RNA-binding activity of the protein and to possibly modulate liquid–liquid phase separation with the viral RNA^29–32^. Thus, given its importance in the various stages of the virus replication cycle, the N protein is considered a promising target for the development of SARS-CoV-2 replication inhibitors.

We report here a novel fluorescence polarization-based HTS assay to identify small molecules that can disrupt the interaction of the N protein with an RNA probe derived from the putative SARS-CoV-2 PS sequence. After screening a customized compound library of approximately 3200 bioactive compounds, several phenolic compounds showing IC_50_ values for RNA probe displacement in the low to sub-micromolar ranges, were identified as potential N protein ligands. L-chicoric acid (CA) was selected for additional binding experiments, which indicated CA binding to both the free and RNA-bound N protein, in the sub-micromolar and micromolar ranges, respectively. Complementary orthogonal biophysical methods, including isothermal titration calorimetry (ITC), ^1^H-saturation transfer difference (STD) and ^1^H^15^N-HSQC NMR experiments, further revealed that CA binds to the CTD. This was confirmed by the crystal structure of the CTD in complex with CA, determined at 1.7 Å resolution, which represents the first CTD structure bound to an exogenous small molecule. In addition, we show that CA inhibited SARS-CoV-2 replication in human lung cells, further suggesting that it may influence N protein-mediated RNA packing in vivo. The latter observation is in line with recently reported models of N protein binding RNA^33^, our ^1^H^15^N-HSQC NMR experiments and the N protein crystal structure binding CA, suggesting an allosteric path for N protein RNA binding inhibition by CA. Taken together, our data provide the first evidence of pharmacologically targeting the SARS-CoV-2 N protein with small molecules, thus paving the way for the rational design of new N protein modulators.

## Results

### Development of an HTS assay to find small molecules capable of inhibiting the RNA-binding activity of the N protein

**Figure 1:**
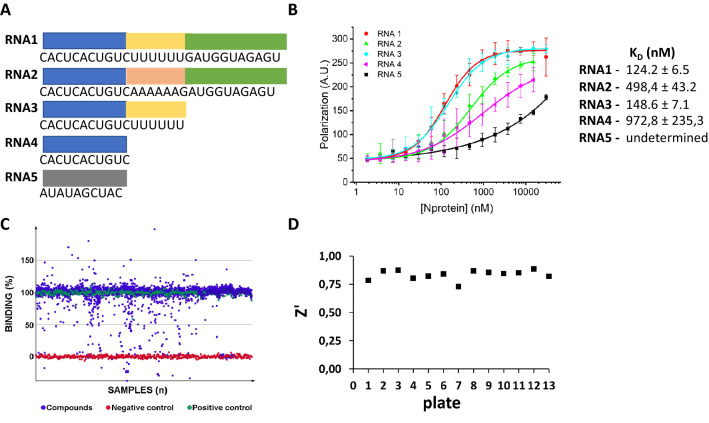
Development of a biochemical assay for the identification of compounds that disrupt the SARS-CoV-2 N protein-RNA interaction. (**A**) Nucleotide sequences of the RNA molecules used as N-protein ligands. The blue, yellow and green regions represent the 3’ arm, the UUUUUU motif and the 5’ arm, respectively. (**B**) FP assay showing the binding affinity curves of the N protein with the FITC-labelled RNA probes 1 to 5 and the corresponding binding equilibrium constants (K_D_) determined by FP. High-affinity binding was observed with RNAs 1 and 3 derived from the putative SARS-CoV-2 PS sequence. The mutated RNA sequences 2 and 4, and RNA5, used as a negative control, show, in comparison, much lower binding affinities. Error bars represent standard deviation of the means from triplicate experiments. (**C**) Scatterplot of HTS results showing the binding percentage of each compound in the library calculated using polarization data from negative (RNA alone) and positive (RNA plus protein) controls. Seventy-eight compounds reduced the binding of the N protein to RNA1 to less than 30%, of which 45 were selected for concentration-dependent assays. Compounds that would promote RNA-N complex formation (activators, binding > 100%) were not further considered. (**D**) Z' values obtained for each assay plate confirming the robustness of the HTS trials.

In order to establish an assay for the identification of potential inhibitors of the N protein RNA-binding activity, we first searched for an N protein target RNA, and the SARS-CoV-2 PS sequence was a candidate. The putative PS of the SARS-CoV-2/SP02/human/2020/BRA strain (nucleotides 19,785–20,364) was identified by sequence alignment to the SARS-CoV and MERS-CoV PS sequences^34,35^. Within this sequence, we selected the CACUCACUGUCUUUUUUGAUGGUAGAGU stem loop (RNA1) as an N protein target (Fig. 1A) because this stem loop is conserved in all SARS-CoV-2 genomes and has an invariable ‘UUUUUU’ motif at the loop.

The RNA-binding activity of the N protein was monitored by a fluorescence polarization (FP) assay using distinct 5’-FITC-labeled RNAs as probes. In addition to RNA1, four other probes were tested (Fig. 1A). In the RNA2 probe (CACUCACUGUCAAAAAAGAUGGUAGAGU) the ‘UUUUUU’ motif was replaced by ‘AAAAAA’, whereas in RNA3 (CACUCACUGUCUUUUUU), the stem loop 5’ arm was deleted. RNA4 (CACUCACUGUC) also lacked the ‘UUUUUU’ motif, while RNA5 (AUAUAGCUAC) served as a scramble negative control (Fig. 1A). As shown in Fig. 1B, optimum binding was observed with the RNA probes 1 and 3 (K_D_ = 124.2 ± 6.5 and 148.6 ± 7.1 nM), respectively, suggesting that the ‘UUUUUU’ motif, but not the hairpin structure, is critical for the interaction with the N protein. Therefore, RNA1 was chosen for the establishment of the protein-RNA dissociation assay used in our HTS trials.

### HTS campaign reveals phenolic compounds as privileged scaffolds that disrupt the binding of the N protein to the target RNA

Following the identification of RNA1 as a high-affinity N protein-binding probe, an FP assay was developed. After checking the assay performance under the screening conditions (Fig. S1A), we conducted an HTS campaign testing a library of ~ 3200 approved drugs and bioactive molecules (Fig. 1C). Differential values of approximately 170 mP were observed between the positive and negative controls (Fig. S1B), resulting in Z’ scores greater than 0.7 for all tested plates (Fig. 1D). Thus, the robustness of the HTS assay allowed us to reliably select hit candidates for concentration–response follow-up experiments.

The first criterium for selecting hit candidates from the primary screening was to choose small molecules that reduced the binding of the N protein to RNA1 to less than 30% (Fig. 1C), which resulted in a list of 78 compounds. From this preliminary list, 33 compounds were flagged due to autofluorescence, interference with the fluorescence or polarization of the free probe. These criteria led us to select 45 hit candidates, representing an overall hit rate of 1.4%.

**Figure 2:**
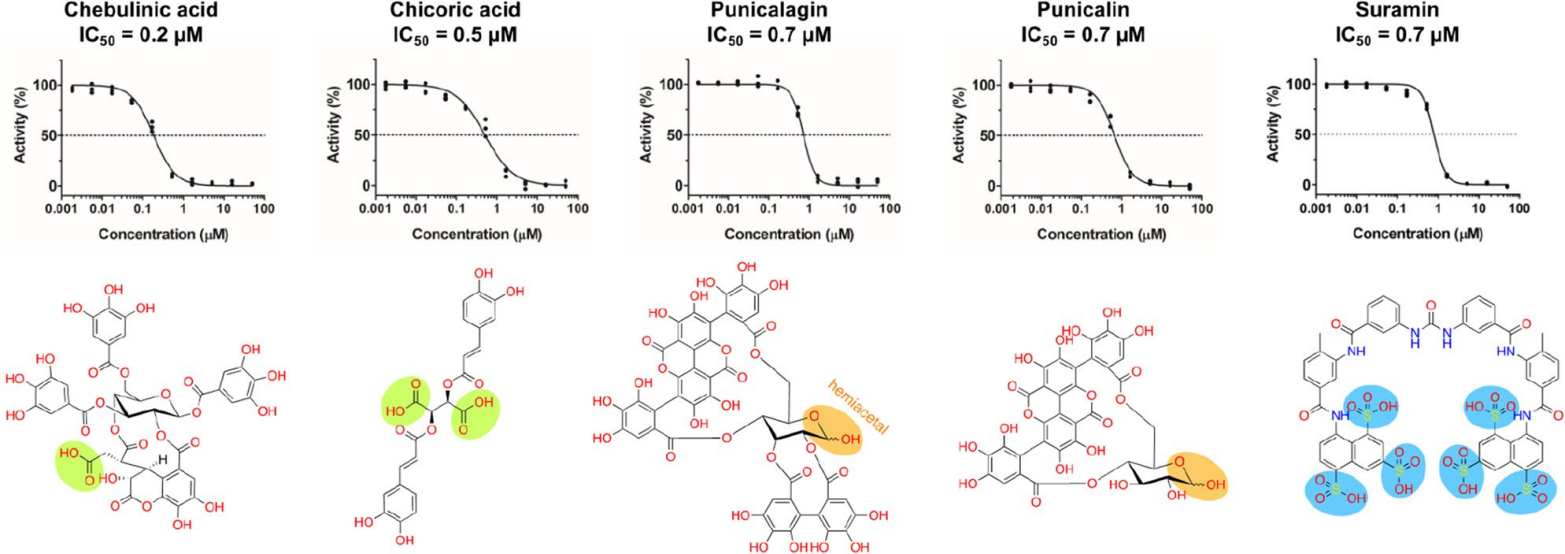
Concentration–response curves and chemical structures of compounds identified as submicromolar N-protein-RNA1 disruptors in the HTS trials. Curves were measured in triplicates, normalized to positive and negative controls and fitted with the log 4-parameter curve in GraphPad Prism. The main non-phenolic functional groups were highlighted as follows: carboxylic acids (green); hemiacetal—in equilibrium with the aldehyde form—(orange); as well as the sulfonic acids (blue) for the polyamide suramin.

To confirm the activity of the hit candidates as inhibitors of the N protein-RNA1 interaction, the selected compounds were subjected to concentration–response experiments (Fig. 2) and 44 of them had their inhibitory activity confirmed (Table 1).

**Table 1:** List of the 44 confirmed hit molecules retrieved from the HTS campaign as potential N-protein ligands.

Compound	IC_50_ (µM)	+ Error*	− Error*
Chebulinic acid	0.2	0.0	0.0
**L-Chicoric acid**	**0.5**	**0.1**	**0.1**
Punicalagin	0.7	0.1	0.0
Punicalin	0.7	0.1	0.1
Suramin	0.8	0.1	0.1
Tannic acid	1.4	0.2	0.2
Chlorophyllin B	2.2	0.2	0.3
Corilagin	2.4	0.5	0.4
**4,5-Dicaffeoylquinic acid**	**3.0**	**0.5**	**0.3**
Methyl Blue	4.2	0.3	0.3
Embelin	5.0	0.9	0.7
Linaclotide	5.5	0.6	0.5
**Isochlorogenic acid A**	**5.5**	**1.0**	**0.8**
Sennoside A	5.7	0.8	0.7
Lusutrombopag	6.0	0.6	0.5
(R)-(-)-Gossypol acetic acid	6.4	0.4	0.4
Idasanutlin	6.5	0.7	0.6
Sulfamerazine	6.5	0.9	0.8
Eltrombopag	7.1	0.9	0.9
Gossypol acetic acid	7.1	0.5	0.4
IOWH-032	7.7	0.9	0.8
Anacardic Acid	7.7	0.9	0.7
Pranlukast (hemihydrate)	7.8	1.1	1.0
Eltrombopag	8.1	1.0	0.9
Succinobucol	8.1	0.6	0.5
RNPA1000	9.6	1.8	1.4
TMC647055	10.0	1.2	1.0
Montelukast	10.3	1.1	1.0
Micafungin	10.4	0.9	0.8
Zafirlukast	10.4	1.6	1.4
Resazurin	10.5	1.7	1.4
Verteporfin	10.8	3.4	2.6
Chlorophyllin A	10.8	1.2	1.0
Surfactin	11.1	1.5	1.3
Pentagalloylglucose	11.6	1.9	1.7
Hexachlorophene	13.0	1.6	1.3
Ertapenem sodium	13.8	1.4	1.3
MK 0893	14.4	1.1	0,9
Butenafine	15.7	1.3	1.2
Oleic acid	15.7	0.9	0.8
Simeprevir	16.5	0.9	0.9
Sofalcone	18.5	1.6	1.5
Bithionol	23.0	5.6	4.6
Gallic acid	23.3	3.3	2.9

Compounds are ranked by their IC_50_ values for blocking the N protein-RNA1 interaction. Phenyl propanoids with dicaffeoyl motif are highlighted in bold.

*Reported errors are deviation from mean value for upper (+ Error) and lower (− Error) limits of 95% confidence intervals.

Many of the compounds that impaired the binding of the N protein to RNA1 are highly polar. Chebulinic acid (CI), CA, punicalagin (PG), punicalin (PL) and suramin (SU) are submicromolar N protein-RNA1 disruptors (Fig. 2). When the N protein was titrated against RNA1 in the presence of CI, CA or PG, we observed that the binding affinity of the N protein to RNA1 was significantly reduced, as revealed by the higher K_D_ values (Fig. S2A), indicating that these compounds inhibit the formation of the N protein-RNA1 complex.

To exclude the possibility that CI, CA or PG could promote protein aggregation, N protein samples were analysed by dynamic light scattering (DLS) in the absence or presence of two-fold molar excess of each compound (Fig. S2B). The results show that, except for the PG, which appears to cause N protein aggregation, the phenolic acids CI and CA at 20 µM final concentration did not significantly change the hydrodynamic radius (Rh) or the oligomeric state of the N protein (Fig. S2B).

### CA binds the CTD and promotes dissociation of the N protein-RNA1 complex

**Figure 3:**
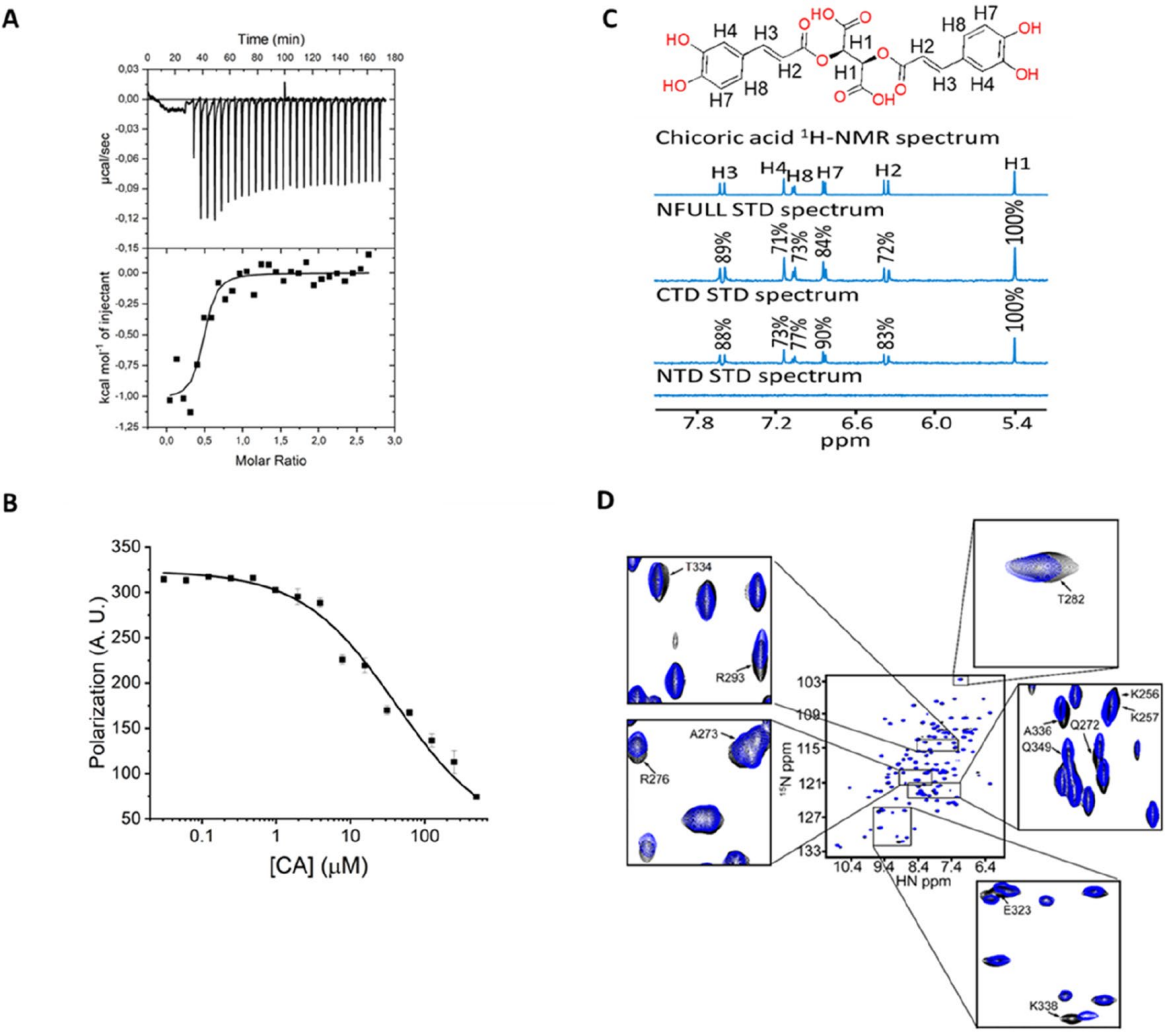
Chicoric acid (CA) is a nanomolar N-protein affinity ligand that binds the CTD and promotes dissociation of the N protein-RNA1 complex. (**A**) ITC assay showing the thermodynamic parameters of interaction between the N protein and CA. The free N protein (20 µM) was titrated against CA (250 µM). The data were fitted to the One Set of Site model, resulting in the following thermodynamic parameters: association constant (K_A_) = 2.27E6 1.3E6 M^-1^ (K_D_ = 0.25 $\pm$ 7.9 E-9 µM); binding enthalpy change (ΔH) = − 953.2 $\pm$ 104.4 cal/mol and binding entropy change (ΔS) = 27.0 $\pm$1.0 cal/mol/deg. The isotherm is representative of three replicates and the values reported are average and standard deviation of the three independent experiments. (**B**) Dissociation curve showing that CA promotes the dissociation of previously formed N protein-RNA1 complex with a K_D_ value of 41.1 ± 11.7 µM. (**C**) One-dimension ^1^H-STD NMR spectra of CA in the presence of free N protein (full-length), CTD or NTD. The relative degree of saturation of each hydrogen atom of CA is mapped onto each spectrum and normalized by hydrogen H1. (**D**) ^1^H-^15^ N-HSQC spectra of free ^15^ N-CTD in the absence (black signals) or presence (blue signals) of CA in a 1:1 protein:CA ratio. Assigned peaks were based on the BMRB entry 50,518. Highlighted peaks correspond to signals that changed by more than 0.02 ppm using the deviation equation Δδ(^15^ N + ^1^H) = [(Δδ^15^N/10)^2^ + (Δδ^1^H)^2^]^1/2^.

Because caffeic acid derivatives have been recently described as potential inhibitors of SARS-CoV-2 infection^36^ and CA, a symmetric dicaffeoyl ester of tartaric acid, exhibits antiviral activity against HIV and hepatitis B virus (HBV)^37–39^, we decided to further investigate the properties of CA as an N protein ligand. First, the binding affinity of CA for the N protein was determined by ITC, which provided the thermodynamic signature of binding, including K_D_, and both the enthalpic and entropic contributions. The results confirmed that CA binds to the N protein with a K_D_ of 250 ± 7.9 nM, and suggested that the CA binding to the N protein is enthalpy driven (Fig. 3A). Given that CA binds to the N protein at nanomolar concentrations, we decided to investigate whether CA could dissociate the N protein-RNA1 complex under conditions that favour N protein-RNA1 complex formation (out of chemical equilibrium). We found that CA promoted the dissociation of previously formed N protein-RNA1 complex with a K_D_ value of 41.1 ± 11.7 µM (Fig. 3B). Although this dissociation constant is much higher than the K_D_ for the CA-N protein interaction, we have to consider that the protein concentration used in this experiment was tenfold higher than the K_D_ value, dislocating the binding equilibrium, and ensuring N protein-RNA1 complex formation.

Finally, the interaction of CA with the N protein was examined by ^1^H-STD and ^1^H-^15^ N-HSQC NMR experiments. The ^1^H-STD NMR technique allows the identification of the binding epitopes of a ligand when bound to a receptor protein. The results confirmed that CA produces clear STD signals which were mapped to its chemical structure (Fig. 3C). Judging by the signal intensity, hydrogen H1 (100% intensity), in CA’s tartaric acid unit, appears to be the most directly involved in N protein binding and thus in closer contact with the protein. In comparison, the H2, H3, H4, H7 and H8 hydrogens, which form the dicaffeoyl units, showed STD signal intensities varying from 70 to 90% (Fig. 3C). Importantly, we found that the STD signals were observed with the full-length protein and CTD, but not with the NTD. This suggests the CA-binding site is located on the CTD of N protein. In addition, ^1^H-^15^ N-HSQC NMR experiments were carried out to further examine the CA-binding to the CTD in solution. This experiment monitors the chemical environment of each protein residue, highlighting the ones that undergo chemical shifts in the presence of the ligand, and therefore are directly involved in ligand binding or affected by the latter event. The ^15^ N-CTD in the absence and presence of CA was inspected by ^1^H-^15^ N-HSQC experiments revealing twelve residues that showed small, but significant chemical shift changes in the presence of CA (Fig. 3D).

Together, these results show that CA is a nanomolar N-protein affinity ligand that binds to the CTD and displaces the RNA from the N protein at micromolar concentrations. In particular, the CA’s tartaric acid unit is involved (H1) and the CTD N-terminal (Lys256, Lys257), dimeric interface (Glu323, Thr334, Ala336, Lys338, Thr282, Gln349) and helices 1–2 (Gln272, Ala273, Arg276, Arg293) regions are affected by CA binding.

### The SR linker is required for RNA1 binding

The results shown above prompted us to test whether RNA1 could also display selective binding affinity for the CTD. Surprisingly, however, we found that RNA1 did not bind to the CTD or the NTD alone in FP assays (Fig. S3A). Binding to RNA1 was nevertheless restored with the NTD-L-CTD protein, in which the NTD and CTD, both lacking their flexible terminal ends, are joined by the SR linker (Fig. S3A).

The FP data showing that the CTD does not interact with RNA1 was further confirmed by NMR experiments that showed no significant chemical shift changes between the ^1^H-^15^ N-HSQC spectra of the ^15^ N-labeled CTD (^15^ N-CTD) in the absence or presence of RNA1 (Fig. S3B), reinforcing the importance of the SR linker for RNA binding.

### The binding mode of CA to the N-protein CTD

To gain important structural information into the binding mode of CA to the N protein, we determined the crystal structure of the CTD in complex with this ligand. CTD crystals belonged to the space group *P* 2_1_2_1_2_1_, with Matthews coefficient^40^ of 2.20 Å^3^ Da^−1^ and solvent content of 44.2%. Datasets for the CTD alone (apo CTD—PDB entry: 7UXX) and in complex with CA (CTD-CA—PDB entry: 7UXZ) were scaled to resolutions of 1.85 and 1.73 Å, respectively. The phases were recovered by molecular replacement, using a previously described SARS-CoV-2 CTD crystal structure (PDB code: 7C22) as the search model^41^. The models were refined to R_work_/R_free_ values of 17.2/20.3% (apo CTD) and 17.4/21.5% (CTD-CA). Data collection and refinement statistics are summarized in Table S1.

**Figure 4:**
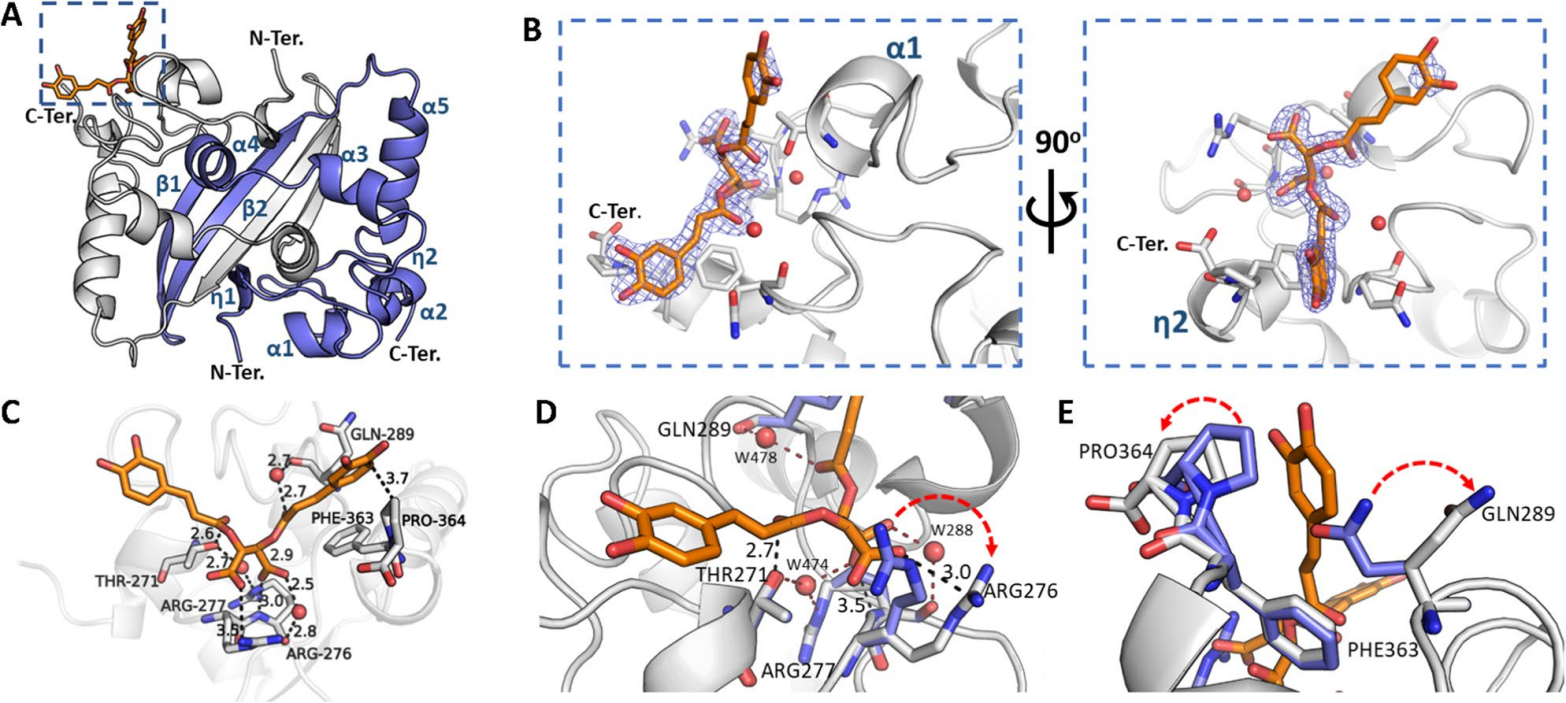
The crystal structure of SARS-CoV-2 N protein CTD binding chicoric acid (CA) reveals a network of polar contacts and structural readjustments to accommodate the symmetric ligand in the CA-N protein binding site. (**A**) Cartoon representation of the N protein CTD dimer crystal structure depicting its secondary structure elements (in blue), including two 3_10_ (η) helices, five α-helices and two antiparallel β-strands and the CA binding site (inset highlighted by the blue dashed square). (**B**) Detailed CA-binding site from the inset of panel (**A**). The CA molecule is represented as sticks (orange) with its electron-density map in blue. CA binds to a shallow pocket formed between α-helices 1–2 and η-helix 2, close to the C-terminus (C-Ter). (**C**) CA atomic interactions with the N protein residues. The CA carboxyl groups are at ideal distances to engage electrostatic interactions and hydrogen bonds with Arg276 side chain (NH1 atom), Arg277 main chain amine and a structural water molecule (W288) stabilized by Arg276 NH2. Thr271 and Gln289 can further position hydrogen bond donors (Thr271O^γ^ and a structural water molecule, W478, stabilized by the Gln289 carbonyl) to engage a symmetric interaction with the carbonyl groups from both caffeoyl units of CA. One of the catechol motifs of CA is well accommodated near the C-terminal Pro364, showing a well-defined electron density (vide panel **B**). (**D**,**E**) Superposition of the CA-binding site in the native N protein CTD (blue sticks, PDB ID 7UXX) and CA-N protein CTD complex (grey sticks (PDB ID 7UXZ) highlighting structural readjustments induced by CA binding (highlighted by red arrows). Figures were generated with Pymol (Schroedinger Inc.). Polar contacts are indicated by dashed lines with the measured distances in Angstroms. Oxygen atoms are shown in red, nitrogen atoms in blue. Water molecules are represented as red spheres.

Six molecules organized as three dimers were found in the asymmetric unit of the CTD. Each protomer in the structures is comprised of five α-helices, two 3_10_ (η) helices and two antiparallel β-strands (Fig. 4A). As reported previously^41^, the CTD dimer is stabilized by extensive hydrogen bonds connecting the β2 strands (Leu331–Ile337) and the interaction of residues from the loop between α-helices 1–2 (Arg277, Gly278, Glu280, Gln283 and Asn285). β-strand 1 and α-helix 4 (Gly316, Arg319 and Ile320) also play an essential role in stabilizing the dimer. In addition, the β-hairpins (Leu331, Leu339), α-helices 3–4 (Ile304, Ala305, Phe314 and Phen315) and α-helix 5 (Phe346–Leu353) form a hydrophobic core stabilized by Van der Waals interactions across the amino acid side chains.

CA binds to a shallow pocket (volume of ~ 190 A^3^ ) formed between α-helices 1–2 and η-helix 2, close to the C-terminus (Fig. 4B). The CA molecule bound to one of the CTD protomers was unambiguously found by inspecting the 2*F*_o_–*F*_c_ electron-density map. Nevertheless, this map only partially covers the CA aromatic ring most exposed to solvent (Fig. 4B).

A close inspection of the structural complex shows that the binding of CA to the CTD is stabilized mainly by polar contacts of the carboxylate and carbonyl groups from CA’s tartarate and caffeoyl units, involving Arg276/Arg277 and three structural water molecules (Fig. 4C). The structure of the complex also shows one CA catechol ring laid inside a hydrophobic canyon formed by η-helix 2, α-helix 2 and the last two C-terminus residues (Phe363 and Pro364).

When the apo and complexed structures were superposed, we observed no significant changes in the overall structures, as judged by the Cα RMSD (0.19 Å deviation). However, we noticed clear conformational changes in the side chains of Phe363, Pro364, Gln289 and Arg276, which contributed to the widening of the CA pocket (Fig. 4D and E). Arg276 had moved outwards, positioning its guanidine group at ideal distances to form electrostatic (NH1) and water-mediated (NH2) hydrogen-bond interactions with CA’s carboxylate group. Arg277’s main chain amine was already at ideal distances to engage in a hydrogen bond with CA’s other carboxylate group. Arg277 N^ε^ atom could further form a water-bridged hydrogen bond with Thr271 (Fig. 4D), which might probably contribute to stabilizing the polar contact network within the CA-N protein binding site.

The catechol ring was also predicted to be important in ligand binding, since Gln289’s polar side chain was turned outwards the pocket, exposing its aliphatic region to the apolar pocket formed by Pro364, Phe363, thus favouring the accommodation of one of CA’s catechol rings, which in turn showed clear electron density (Fig. 4E). Furthermore, Arg293 side chain showed double conformation, one of them in clash distances to the Gln289 side chain. The second catechol ring of CA is more solvent exposed, however under pi-pi interactions distances to the Gln272 side chain carboxyl group.

In addition to the crystallographic observations, an initial structure–activity relationship (SAR) analysis could be performed based on 16 compounds assessed in our screening efforts containing caffeoyl substructures, further supporting CA binding to this pocket on the N-protein CTD (Fig. S6).

### Structural consequences of CA binding for N protein function

**Figure 5:**
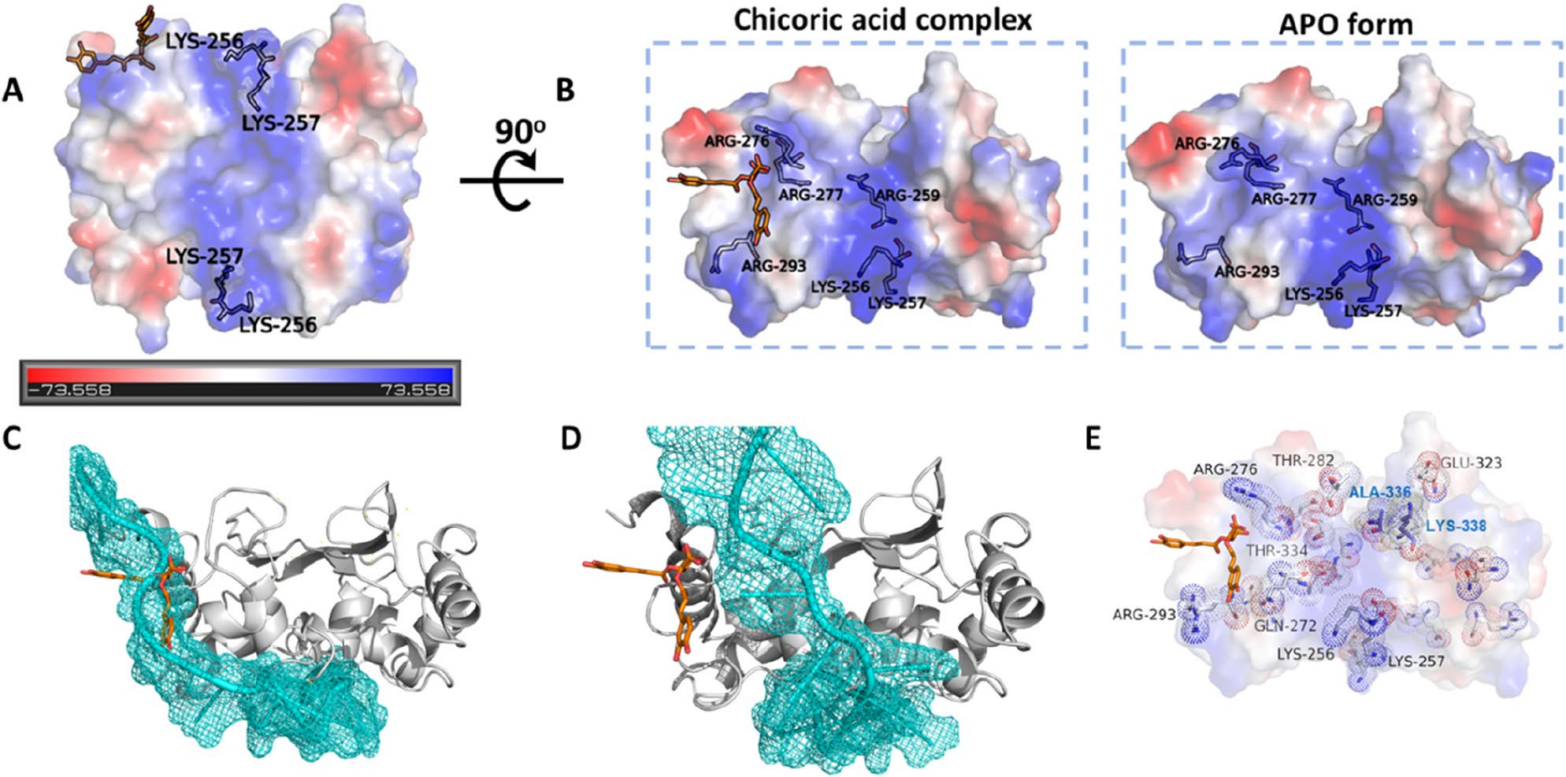
Chicoric acid (CA) binding to SARS-CoV-2 N protein CTD changes the topology and electrostatic character of the putative RNA-binding region with implications to RNA binding. (**A**) N protein CTD electrostatic potential with red and blue colors indicating negative and positive potentials, respectively (please refer to the electrostatic scale bar). The positively charged groove, formed by Lys256, Lys257, Lys259 Lys261 and Arg262, is thought to contribute for RNA binding. (**B**) The positively charged groove depicted in panel A extends towards the CA-binding pocket through Arg259, Arg276, Arg277 and Arg293. The eletrostacic potentials calculated from the CA-N protein CTD (PDB ID 7UXZ) and native N protein CTD (PDB ID 7UXX) crystal structures are shown. Figures and electrostatic potential were generated with Pymol (Schroedinger Inc.). (**C**,**D**) Structural alignment between the CTD-CA complex and a model of the N protein complexed with an RNA. The CTD chain (grey cartoon) complexed with CA (orange sticks) was superposed to two CTD chains from a N protein model complexed with RNA (cyan), which shows two distinct RNA-binding modes^33^. In the first alignment (**C**), the CA molecule fully occupies the predicted RNA-binding site, whereas in the second alignment (**D**), the CA site is near the RNA-binding site. In both scenarios CA should interfere in N protein binding to RNA. (**E**) CTD residues that underwent chemical shift changes (shown as sticks and dots) upon CA binding in the HSQC experiment, here mapped on the CTD crystal structure binding CA. Residues discussed in the text are labelled. Ala-336 and Lys-338 (labelled in blue) are form the second protomer of the CTD dimer.

The CTD structure in its apo form shows an electrostatic potential distribution that is conserved amongst all N proteins from the *Coronaviridae* family^22,41^, with a major positively charged groove located on one side of the dimer surface (Fig. 5A). This region—which is flanked by Lys256 and Lys257 from both protomers in a CTD dimer and further composed by Lys259, Lys261 and Arg262—is thought to contribute to RNA binding^33,42,43^. We observed that this positively charged groove extends towards the CA-binding pocket through Arg259, Arg276, Arg277 and Arg293 (Fig. 5B). Notably, the binding of CA to the CTD not only changes the topology of this region, but also disrupts the continuity of such potential RNA-binding region, with the possibility to affect N protein binding to RNA in a direct (Fig. 5C) or indirect way (Fig. 5D).

The CTD residues that undergoes chemical shifts changes upon CA binding, according to our HSQC experiments (Fig. 3D), were further mapped on the crystal structure of the CTD binding CA (Fig. 5E). This data gives further insights on how CA might affect RNA binding to the N-protein, showing an allosteric path connecting the CA binding site to the putative RNA-binding groove (Fig. 5A) and its extension (Fig. 5B). In this map, Arg276, which interacts with the carboxylic acid of CA, connects Thr282 in the dimeric interface and can potentially affect Ala336 and Lys338 in the second protomer of the CTD dimer, affecting the extension of the RNA-binding groove. Arg293 and Gln272, displaced by the phenolic rings of CA upon binding, are in close proximity to the CTD N-terminal region, Lys256 and Lys257. The latter residues flank the main positively charged surface of the CTD (the putative RNA-binding groove) and its extension towards the CA binding site side of the CTD. In addition, Lys256 and Lys257 are the first ordered residues of the CTD, connecting the CTD to the NTD-CTD linker, this linker being essential for many N-protein functions^29–32^, including RNA binding as shown here (Fig. S3).

### CA displays anti-SARS-CoV-2 activity in in vitro cell assays

**Figure 6:**
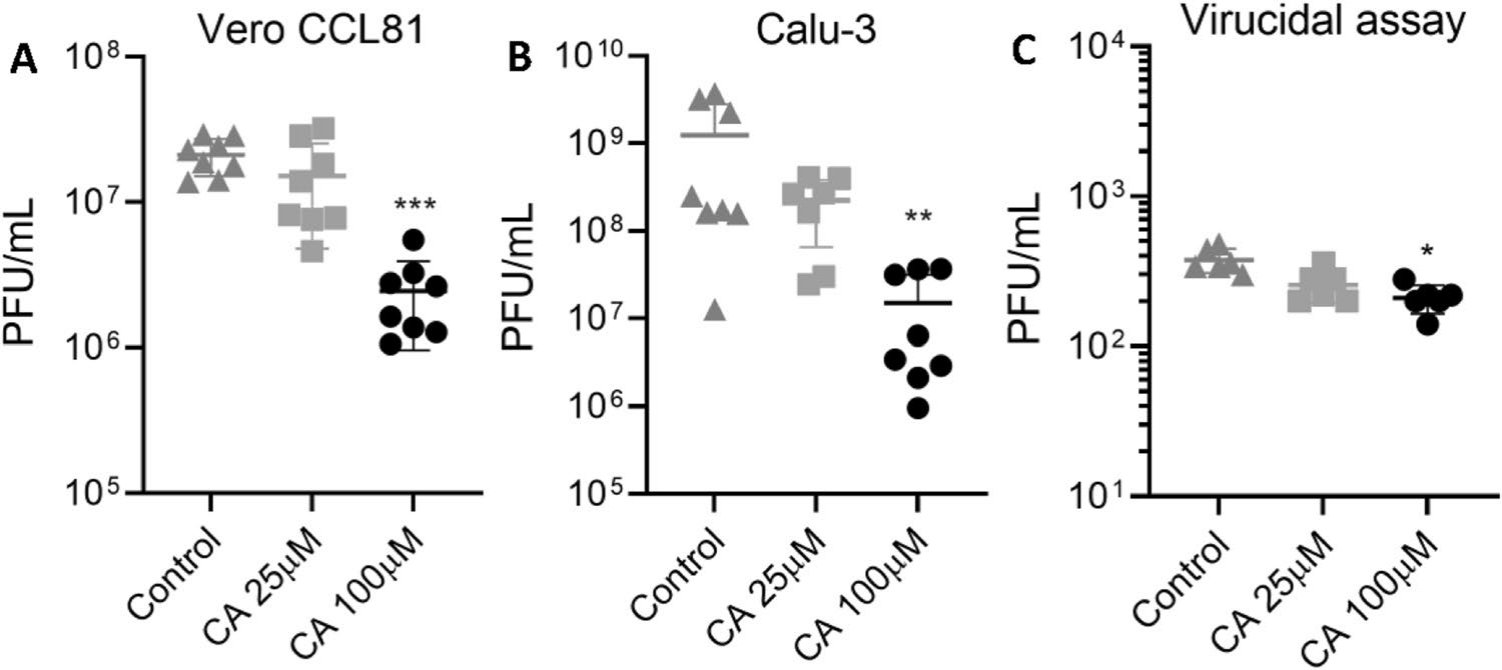
Anti-SARS-CoV-2 activity of CA in cell assays. In vitro replication assays performed in Vero CCL81 (**A**) and Calu-3 (**B**) cells. Cells were infected with SARS-CoV-2 for 1 h and later incubated with fresh media containing CA at 25 µM or 100 µM. The viral load was quantified by the plaque assay (PFU/ml) in the cell supernatants collected 48 h post-infection. (**C**) Virucidal assays performed in Vero CCL-81 cells, where SARS-CoV-2 particles were incubated with CA at 25 µM or 100 µM for 1 h and then used to infect the cells for 1 h. The viral load was quantified using the plaque assay to assess the infectious viral progeny. DMSO at 0.2% final concentration was used as control. *p* ≤ 0.05 (*), *p* ≤ 0.01 (**), *p* ≤ 0.001 (***). Results are expressed as individual values (n = 8 for replication assay and n = 4 for virucidal assay) with mean +/− standard deviation.

After confirming CA as an N protein ligand with potential implication on its RNA binding function, we tested if CA could inhibit SARS-CoV-2 infection in vitro. For this, Calu-3 and Vero CCL81 cells were seeded into 24-well plates at 2 × 10^5^ and 2.5 × 10^5^ cells/well, respectively, and infected with SARS-CoV-2 at a multiplicity of infection (MOI) of 1. CA was added to the cell culture medium after infection at 25 and 100 µM final concentrations, whereas DMSO at 0.2% was used as a vehicle control. Assessment of the viral load in the cell culture supernatants collected 48 h post-infection indicated that CA presented antiviral activity at 100 µM only, relative to untreated (vehicle) control. CA treatment caused tenfold and 100-fold reductions in infectious viral load in Vero CCL81 (*p* < 0.001—Fig. 6A) and Calu-3 (*p* < 0.01—Fig. 6B), respectively. Both cell lines remained viable when treated with CA or DMSO at the tested concentrations (Fig. S5). In addition, CA displayed only a slight virucidal activity against SARS-CoV-2 (*p* < 0.05—Fig. 6C), indicating that the observed antiviral effect of CA cannot be attributed to direct action on viral particles. These results thus show that CA inhibits SARS-CoV-2 replication in cell culture at micromolar concentrations, which is consistent with the K_D_ values for the N protein-RNA1 complex dissociation (Fig. 3B).

## Discussion

Despite the great success of newly developed vaccines to prevent contagion and severe forms of infection by SARS-CoV-2, infections are still present, and new waves of contamination by viral variants are recurrent. In this sense, antiviral drugs are needed to improve patient recovery and to prevent disease progression, especially to at-risk patients. The development of such antiviral drugs, in many cases, requires knowledge of the structure and function of potential viral targets and the discovery and characterization of small-molecule modulatory binding sites in these proteins, that afford for structure-based drug design (SBDD) approaches^44^.

In this work, we describe a novel fluorescence-based high-throughput screening assay that allows the identification of small molecules that interfere with the RNA-binding activity of the SARS-CoV-2 N protein, the most abundant viral protein expressed in host cells, and which plays fundamental roles in transcriptional regulation and virus assembly^20,22–26,29–32^. We further characterized the top hits, especially chicoric acid (CA), using a cascade of biophysical assays (Fig. 3), and solved, for the first time, the crystal structure of the N protein CTD binding a non-endogenous ligand, CA, further revealing a new modulatory site in the SARS-CoV-2 N protein (Fig. 4).

By screening a customized library of bioactive small molecules (Fig. 1), highly polar compounds stood out, highlighting polyphenols (ellagitannins, CI, PG and PL), a diester of tartaric acid (CA) and a polysulphonated naphthylurea (suramin, SUR)—Fig. 2. The latter compounds were capable of disrupting the interaction of the full-length SARS-CoV-2 N protein with an RNA probe derived from the SARS-CoV-2 PS sequence (RNA1) in the submicromolar range (Fig. 2).

In accordance with the literature, the antiparasitic drug suramin is reported to inhibit SARS-CoV-2 infection in cell culture at 20 µM, by interfering with early steps of virus replication^45^*.* The natural ellagitannins CI, PG and PL have already been reported to exhibit diverse biological properties^46^, including antiviral activity against multiple human viruses with in vitro potencies in the low micromolar range^47–49^. Other polyphenols, like catechin gallate and gallocatechin gallate, have also been shown to reduce the binding affinity of an RNA oligonucleotide to the N protein of SARS-CoV, causative of the 2003 coranavirus outbreak, on a biochip assay^50^.

Chicoric acid (CA), a symmetric dicaffeoyl ester of tartaric acid, was highlighted in the present study as a new class of N protein modulator and one of the most potent hit compounds identified in our HTS trials. Importantly, the binding of CA to the N protein was further characterized by biophysical methods (Fig. 3) and the crystal structure of CA binding to the SARS-CoV-2 N protein CTD could be determined at 1.7 Å resolution (Fig. 4).

Although polyphenols have been shown to interfere with coronavirus N proteins before^50^ and compound PJ34 (SARS-CoV NTD^51^) and GTP (SARS-CoV-2 CTD) interacted with N proteins, our data show that CA is a new class of N protein ligand that binds to a new modulatory site on the SARS-CoV-2 N-protein CTD. Importantly, the CA-binding site is conserved in SARS-CoV and partially conserved in MERS N proteins (Fig. S4). To our knowledge, this is the first description of a non-endogenous SARS-CoV-2 N protein ligand and the first report of this modulatory ligand binding site on a coronavirus N protein.

We present evidence that the N protein CTD is sufficient for interaction with CA, as revealed by the ^1^H-STD NMR experiment carried out with the full length, CTD and NTD SARS-CoV-2 N protein (Fig. 3C). HSQC (Fig. 3D) and crystallographic data (Fig. 4) further confirmed that CA binds to the SARS-CoV-2 N protein CTD in a shallow pocket formed mainly by helices 1–2, being sandwiched by the CTD’s C- and N-terminus. This site is connected to the positively charged RNA binding groove by Lys256 and Lys257, which in turn flank the RNA binding groove, and its extension towards the CA binding site side of the CTD (Fig. 5). CA binding to N protein is stabilized mainly by a polar contact network involving ionic interactions of CA’ symmetric carboxylates with the N protein’s arginines and water-mediated hydrogen bonds of its caffeoyl carbonyl groups. The catechol rings of CA are further involved in ligand binding and induce conformational changes in the N protein, by docking to hydrophobic pockets formed by glutamine residues in both sides of the CA-binding site (Fig. 4). The two carboxylate and two caffeoyl ester moieties seem important for binding to the N protein (Figs. 3C, 4 and S6) and to induce conformational changes (Figs. 3D and 5) that might affect its RNA binding function.

The functional effects of CA binding to the SARS-CoV-2 N protein could be further explored in the present work. The CA-binding site in N protein is located adjacent to the main positively charged groove thought to interact with the RNA^42,43^ (Fig. 5). Notably, the binding of CA to the CTD not only alters the topology but also the charge distribution of this region, which might explain why CA disrupts the N protein-RNA1 complex in solution. This hypothesis is additionally supported by a structural model of the SARS-CoV-2 N protein complexed with an RNA molecule, which we have recently reported^33^. This structural model presupposes two possibilities: one in which the CA-binding site fully overlaps with the RNA interaction site (Fig. 5C), and the second where the CA site is near the RNA site (Fig. 5D). The second scenario seems more plausible, because, as we showed here, the CTD alone did not interact with RNA1 and the presence of the SR linker was critical for RNA1 binding (Fig. S3). In both scenarios, however, it can be anticipated that the binding of CA to the CTD could preclude RNA interaction, therefore modulating the essential N protein functions related to SARS-CoV-2 genomic RNA binding. In addition, our HSQC experiments showed that the binding of CA to the CTD causes chemical shifts in residues of the CA binding site (e.g. Arg276, Arg293 and Gln272) and also in residues of the CTD that are not directly involved in CA binding (Fig. 3D and Fig. 5E). Some of these residues might affect the stability of the NTD-CTD linker (e.g. Lys256 and Lys257) in the full-length protein and the CTD’s dimeric interface (e.g. Thr282, Thr334, Ala336, Lys338). Importantly, Lys256, Lys257 and Lys338 are also placed in the CTD’s positively charged grooves, which potentially interact with SARS-CoV-2 RNA (Fig. 5E).

CA showed binding affinities for the N protein of 0.25 μM (Fig. 2); nevertheless, the CA equilibrium constant for the N protein-RNA1 complex competition in chemical equilibrium was 0.5 μM (Fig. 2). Such dissociation constant is consistent with the higher N protein binding affinity exhibited by RNA1, and with the notion that RNA1 has a much larger interaction surface than CA. Interestingly, out of the chemical equilibrium, when complex formation is induced by higher protein concentration, the complex achieves 50% of dissociation in the presence of ~ 40 µM of CA (Fig. 3B). This experiment simulated a context of active viral infection, in which viral proteins and RNA are present in relative high concentrations, promoting complex formation and viral packing. These data, aligned with the reported low cell permeability of CA^52,53^, also help to explain the relatively high CA concentration required to significantly inhibit SARS-CoV-2 replication in human cells (Fig. 6). Although further optimization of CA as an antiviral agent is needed, our data offer the structural basis for the rational design and development of novel antiviral drugs targeting the SARS-CoV-2 N protein, an essential and yet underexplored target of coronaviruses.

## Methods

### Protein expression and purification

The gene sequence corresponding to the full-length SARS-Cov-2 N protein (GenBank QIG56001.1) was amplified from SARS-CoV-2 RNA and cloned into a pET28a-TEV vector for the expression of a 6xHis-fusion protein, as previously described^54^. The NTD (residues Q43-E174) and CTD (residues S250-P364) fragments were amplified from the full-length N construct using primers forward (NTD-F 5’-AACGTGGATCCCAAGGTTTACCCAATAATACTG-3’, CTD-F 5’CTAAGGGATCCGCTGCTGAGGCTTCTAAGAAG3’) and reverse (NTD-R 5’-ACTGCCGCGGCCGCTTTATTCTGCGTAGAAGCCTTTTGG-3’, CTD-R 5’CTTTTTAGCGGCCGCTTATGGGAATGTTTTGTATGCGTC3’), respectively, and inserted into the *BamH*I/*Not*I sites of a pET-SUMO vector (Invitrogen), carrying a SUMO sequence at the N-terminus. The expression vectors were used to transform *Escherichia coli* BL21 (DE3) cells (Novagen, USA). Freshly transformed cells were grown in LB-kanamycin (50 μg/mL) medium to OD600nm 0.8 at 37 °C. The temperature of the cultures was lowered to 25 °C (full-length N) or to 18 °C (NTD and CTD), and protein expression was induced with 0.1 mM (full-length N) or 0.5 mM (NTD and CTD) IPTG for 16 h at the respective temperatures. Cells were harvested by centrifugation (4000 × g, 10 min) and stored at − 80 °C. To remove nucleic acids of bacterial origin, the proteins were purified under denaturing conditions using urea and high salt concentration^29^. Frozen cell pellets were thawed and resuspended in buffer A (50 mM sodium phosphate, pH 7.6, 500 mM NaCl, 10% glycerol, 20 mM imidazole, 6 M urea) and lysed by sonication on ice. Lysed cells were centrifuged at 18,000 × g for 40 min at 4 °C to remove cell debris and the supernatants were applied onto a HisTrap FF 5 mL column (GE healthcare) pre-equilibrated with buffer A. After washings, proteins were eluted in ten column volumes of buffer B (50 mM sodium phosphate, pH 7.6, 500 mM NaCl, 10% glycerol, 500 mM imidazole, 3 M urea). Fractions containing the protein of interest were pooled and dialyzed against buffer C (50 mM sodium phosphate, pH7.6, 500 mM NaCl, 10% glycerol) overnight at 4 °C. Except for the N-full construct, the recombinant proteins (NTD and CTD contructs) were cleaved with the appropriate TEV and SUMO proteases. Cleaved tags were removed by reverse affinity chromatography using buffer A and B without urea. Protein fractions were concentrated and fractionated on a size exclusion Superdex 200 16/600 (full-length N) or Superdex 75 16/600 (NTD and CTD) column, previously equilibrated with buffer C.

For crystallization tests, the CTD was purified using Turbonuclease from *Serratia marcescens* (Sigma, USA). Briefly, bacterial cells after IPTG induction were suspended in lysis buffer (50 mM Tris HCl pH 8.0; 1 M NaCl, 5% glycerol, 1 mM β-mercaptoethanol) containing 200 units of Turbonuclease and lysed by sonication as described above. Lysed cells were centrifuged, and the supernatant was applied onto a HisTrap FF 5 mL column pre-equilibrated with buffer D (50 mM Tris HCl pH 8.0, 500 mM NaCl, 5% glycerol, 1 mM β-mercaptoethanol). Bound proteins were eluted using the same buffer containing 500 mM imidazole. The eluate was dialyzed against buffer D overnight at 4 °C. After SUMO cleavage and reverse affinity chromatography, the proteins were fractionated on a Superdex 75 16/600 column pre-equilibrated with buffer E (20 mM Tris HCl, pH 8.0, 100 mM NaCl, 1 mM β-mercaptoethanol).

The quality of all protein preparations was verified by SDS-PAGE and dynamic light scattering (DLS). In addition, UV absorbance at 260/280 nm was used to estimate the amount of nucleic acid in the protein samples. Only protein samples with a monodisperse character and a 260/280 nm ratio of 0.5–0.6 were used in the experiments described below.

### Fluorescence polarization assay

Chemically synthesized RNA probes 5’-labelled with fluorescein isothiocyanate (FITC) and purified by HPLC were obtained from Thermo Scientific (USA). Probe sequences were as follows: RNA1 (5’-CACUCACUGUCUUUUUUGAUGGUAGAGU-3’), RNA2 (5’-CACUCACUGUCAAAAAAGAUGGUAGAGU-3’), RNA3 (5’-CACUCACUGUCUUGUUUGAUGGUAGAGU-3’), RNA4 (5’-CACUCACUGUCUUUUUU-3’), RNA5 (5’-CACUCACUGUC-3’) and scramble control RNA6 (5’-AUAUAGCUAC-3’).

Fluorescence polarization (FP) assays were used to determine the binding affinity of the N protein to the RNA probes, solubilized in 50 mM sodium phosphate buffer (pH, 7.6). Purified N protein from 2.5 nM to 15 µM in 50 mM sodium phosphate buffer, 100 mM NaCl (pH, 7.6) was mixed with each RNA at 10 nM final concentration, in 384-well plates. FP data was acquired using a ClarioStar microplate reader (BMG LabTech), with excitation and emission wavelengths set to 485 and 530 nm, respectively. Affinity binding curves were fitted to a Hill1 model using the OriginPro software.

### Hight-throughput screening assays

A customized library with 3215 nonredundant compounds from the collections ‘FDA-approved’, ‘anti-COVID’, ‘anti-infection’ and ‘anti-virus’, was purchased from MedChemExpress (NJ, USA). The library, in 384-well plates, was diluted to 1 mM concentration in dimethyl sulfoxide (DMSO) and stored at − 20 °C. Columns 1, 2, 23 and 24 of all microplates were filled with DMSO for screening controls as described below.

Binding of RNA1 to the N protein was monitored by FP, as described above. Screenings were performed in 384-well, flat bottom, black polypropylene microplates (Greiner #781,289), using the binding buffer supplemented with 0.01% triton X-100, in a final volume of 25 µL. The final concentration of RNA, N protein, library compound and DMSO were 10 nM, 500 nM, 20 µM and 2% (v/v) respectively. Initially, the assay plates were filled with the RNA probe solution (19.5 µl) using a MultiDrop dispenser (Thermo Fisher) and the compounds (0.5 µl) were transferred from the library to the assay plates in a Janus-MDT liquid handler platform (PerkinElmer). FP measurements were performed at this stage to detect possible interference from library compounds. The N protein (5 µl) was then transferred to all wells of the assay plates using the MultiDrop dispenser, except for columns 1 and 24 which received buffer, RNA and DMSO only, and were used as negative controls (low control, free probe). On the other hand, columns 2 and 23 received buffer, RNA, protein and DMSO, and were considered as positive controls (high control, bound probe). After adding the protein to the assay mix, the plates were incubated at room temperature for 30 min before FP was measured. mP values of positive (100% binding) and negative (0% binding) controls were used for sample data normalization.

### Concentration − response curves and IC_50_ determination

To confirm the activity and evaluate the potency of selected hit candidates, hit compounds were subjected to dose–response assays. Except for compound concentration, the assay conditions were as described above, where 0.5 µL of the compounds were transferred to the assay plates generating a concentration gradient from 1.8 nM to 50 µM. The concentration–response assays were performed in triplicates and after acquisition of the FP data, the percentage of inhibition for each test compound was calculated as follows: % inhibition = (high control average − read value)/(high control average − low control average) × 100. Normalized data was fit to the log4 parameters equation with variable slope to extract the *IC*_50_ values with GraphPad Prism 9 (GraphPad LLC, v. 9.3.1).

### Biophysical analysis and hit confirmation

Aggregation assays were performed with the full-length N protein diluted to 10 µM in 50 mM sodium phosphate buffer, 100 mM NaCl (pH, 7.6), with subsequent addition of 20 µM of each test compounds or 1% DMSO used as diluent. The same assay was performed with the protein at 2.5 µM in the presence of 500 µM CA. Samples were evaluated by DLS in a ZetaSizer NanoS (Malvern) equipment, at 10 °C, using default parameters set by the equipment.

The affinity of RNA1 for the N protein in the presence of selected hit compounds was inspected by FP. Serial dilutions of the protein:compound mixtures at a 1:2 ratios were prepared in 50 mM sodium phosphate buffer, 100 mM NaCl (pH, 7.6). RNA1 at 10 nM was added to the serial dilutions and FP measurements were performed as described above.

To determine the amount of CA required for N protein-RNA1 dissociation in conditions which complex formation is induced by dislocating binding equilibrium was performed. For this, purified N protein at 2.5 µM (concentration 10 times higher than the KD for N protein RNA, to ensure that complex formation is induced) in 50 mM sodium phosphate buffer, 100 mM NaCl (pH, 7.6) was incubated 10 nM RNA1 at 4 °C for 60 min prior to the addition of increasing amounts of CA up to 500 µM. The FP data were acquired as described above and the affinity binding curves were fitted to a Hill1 model using the OriginPro software.

The dissociation constant and thermodynamic parameters for the N protein-CA interaction were determined by ITC using a VP-ITC calorimeter (Malvern). Purified N protein was dialyzed against 50 mM sodium phosphate buffer, 500 mM NaCl (pH, 7.6), overnight at 4 °C, and further diluted to 20 µM. The dialysis buffer was used to prepare CA at 250 µM. Both solutions, from cell and syringe, were prepared in the presence of 0.25% DMSO. CA was titrated against the protein solution (10 µL injections) at 20 °C with 300 s intervals. CA titrations against the buffer and buffer titrations against the protein solution were performed as controls. The isotherm curves, after subtracting the controls, were analysed using the Microcal Origin software provided with the equipment, and the data were fitted to One Set of Sites model.

### Protein crystallization and X-ray data collection

Freshly prepared CTD samples at 8 mg/mL were subjected to hanging-drop vapor diffusion crystallization trials performed in 24-well VDX plates at 18 °C using Hampton Crystal Screen HT™ solutions. After optimizing the crystallization conditions, CTD crystals were obtained within three days in 100 mM Tris -HCl (pH 8.3), 30% PEG 4000, 0.2 M sodium acetate, with 2 µL drops (1 µL protein/1 µL reservoir solution) and 300 µL reservoir solution. Protein–ligand crystals were grown within three weeks under the same crystallization condition with a reservoir solution containing 4 mM CA. Protein crystals were cryoprotected by rapid soaking in reservoir solution containing 25% glycerol and flash-cooled in liquid nitrogen. X-ray diffraction data were collected under cryogenic conditions (100 K) at 1.327 Å and 0.977 Å wavelength at the Manacá beamline (macromolecular micro and nano crystallography)^55^ of Sirius, the Brazilian synchrotron light source (LNLS, Campinas, Brazil), using a PILATUS 2 M detector placed 145 mm from the crystal. The X-ray data were collected using a fine ϕ-slicing strategy, rotated through 360° with a 0.1° oscillation range per frame.

### X-ray data processing and structural determination

X-ray diffraction data were automatically processed with XDS^56^ using the Manacá Automatic Processing Pipeline (ManacáAutoProc) and analyzed and scaled using Pointless, Matthews and Scala from CCP4 package^40,57,58^. The phases of the datasets were determined by molecular replacement with Molrep^59^ and Phaser^60^ using the SARS-CoV-2 CTD crystal structure (PDB code: 7C22) as the search model. The atomic structures were refined using REFMAC5^61^, ligands had their geometry restraint information generated for refinement by eLBOW^62^ and then modelled using COOT^63^. All figures were generated using PyMOL.

A close inspection of symmetry related molecules reveals an impediment to chicoric acid interaction to other N-CTD protomers in the crystal. This close contact refers to the symmetric N-CTD loops GLU280–GLN283 and VAL324–GLY328. All three dimers in the asymmetric unit present this impediment in one of the protomers due the crystal packing, resulting in the identification of only one chicoric acid binding site in the asymmetric unit of the reported crystal structure.

The volume of the CA binding site was estimated using parKVFinder software^64^ using the box adjustment mode around the CA molecule with the following parameters: probe in of 1.4 Å, probe out of 12 Å and removal distance of 0.5 Å.

### NMR experiments

All NMR spectra were obtained using an Agilent DD2 500 MHz spectrometer or Varian Inova 600 MHz spectrometer both equipped with a 5 mm triple-resonance probe and a Z pulse-field gradient unit at 298 K. The STD experiments were performed with 400 μM CA and 4 μM N protein samples dissolved in 80 mM sodium phosphate buffer, pH 7.4, prepared with deuterated water. The 1D ^1^H-STD spectra were obtained by subtracting the saturated spectra (on- resonance) from the reference spectra (off-resonance), which was automatically performed by phase cycling using the dpfgse satzfer pulse sequence implemented in the VNMRJ software (Agilent). The spectra were acquired using 2048 scans with a selective irradiation frequency of the protein at0 − .5 ppm (on-resonance) and 30 ppm (off-resonance). Forty G-shaped pulses of 50 ms separated by 1 ms delays were applied to the samples. The total length of the saturation train was 2.5 s. A T_2_ filter was applied to eliminate all protein background. The off-resonance spectra were used as reference spectra and were acquired with 1024 scans keeping all other parameters equal to the 1D ^1^H-STD-NMR spectra. For the group epitope mapping analysis, the STD enhancements (A_STD_) were determined by the integrals of individual protons of the ligands in the 1D ^1^H-STD-NMR spectrum (I_STD_) divided by the integral of the same signals at the reference spectrum (I_0_) and multiplied by the excess ratio of ligand to protein concentration ([L]/[P]) according to Eq. 1.

1 $$A_{STD} = \frac{{I_{STD} }}{{I_{0} }} \times \frac{\left[ L \right]}{{\left[ P \right]}}$$

### ^15^ N-HSQC protein titration

^15^ N-labelled CTD (^15^ N-CTD) samples were dissolved in 50 mM sodium phosphate buffer, pH 7.6, with 500 mM NaCl and 10% (v/v) deuterated water, to a final concentration of 1.0 mM. Water suppression was achieved using the WATERGATE method^65^. Protein–ligand titrations were performed by direct addition of small aliquots of CA or RNA1 to 5 mm Shigemi tubes containing 300 μL of ^15^ N-CTD such that the ligand:protein ratios were 0:1, 0.1:1, 0.2:1, 0.3:1, 0.5:1 and 1:1. The binding of ligands was characterized by changes in ^15^N-HSQC chemical shift peaks. Chemical shift differences (Δδ average) between the ^15^ N-HSQC spectra of the protein in the absence and presence of the ligands (1:1 ratio) were determined by the equation Δδ(15 N + 1H) = [(Δδ15N/10)2 + (Δδ1H)2]1/2^66^, and deviations greater than 0.02 ppm were considered as significant changes.

### In vitro anti-SARS-CoV-2 activity

Vero CCL81 cells (African green monkey kidney cell line—BCRJ, # 0245) were cultivated in DMEM medium supplemented with 10% fetal bovine serum (FBS), 1% L-glutamine and 1% penicillin/streptomycin. Calu-3 cells (Human lung cell line—ATCC, # HTB-55™) were cultivated in DMEM/F12 (1:1, v/v) medium supplemented with 20% FBS, 1% L-glutamine and 1% penicillin/streptomycin. Both cell lines were grown at 37 °C with 5% CO_2_.

Antiviral assays were performed with the HIAE-02 SARS-CoV-2/SP02/human/2020/BRA strain (GenBank accession MT126808.1) kindly provided by Prof. Edison Luiz Durigon (USP-SP, Brazil). Virus stocks were propagated in Vero CCL81 cells in 75 cm^2^ flasks. After 30–36 h of growth, culture supernatants were centrifuged to remove cell debris and stored at − 80 °C. All assays involving infectious virus were performed in the BSL-3 unit of the Emerging Viruses Laboratory (LEVE) at the State University of Campinas, Brazil.

Cell viability in Vero CCL-81 and Calu-3 after CA treatment was measured by the MTT [3-(4,5-dimethylthiazol-2-yl)-2,5-diphenyl tetrazolium bromide] (Sigma–Aldrich, USA) method. Briefly, CA diluted in 10% DMEM at 25 and 100 µM final concentration was added to the confluent monolayer of cells grown in 24-well plates. After 48 h growth, the medium was replaced by fresh DMEM containing MTT (200 µg/mL) and cells were incubated for 3 h. DMSO was used to solubilize the formazan crystals and cell viability was measured by OD at 492 nm. The results were expressed according to the equation (T/C) × 100%, where T and C represented the mean optical density of treated and control, respectively. DMSO at 0.2% (v/v) in DMEM medium was used as the vehicle control.

To evaluate the activity of CA on SARS-CoV-2 replication, Calu-3 and Vero CCL-81 cells were seeded into 24-well plates at 2 × 10^5^ and 2.5 × 10^5^ cells per well, respectively. The antiviral activity was determined at multiplicity of infection (MOI) of 1. The confluent monolayer of cells was incubated with the virus for 1 h, after which the culture medium was replaced by fresh medium containing CA at 25 and 100 µM final concentration. Culture supernatants were harvested 48 h after virus inoculation and viral load was determined by plaque assay.

### Plaque assay

Vero cells were seeded into 24-well plates and incubated for 1 h with the supernatants from the antiviral assays, serially diluted to 10^−6^. After virus incubation, cells were overlaid with semi-solid medium (1% w/v carboxymethylcellulose in DMEM supplemented with 5% FBS) and incubated for 4 days. After removal of semi-solid medium, cells were fixed with paraformaldehyde 4% and plaques were visualized after 1% methylene blue staining. Viral lysis plaques were counted, and the results were expressed as viral plaque forming units (PFU) per mL of sample.

## Supplementary Information

Supplementary Information.

## Data Availability

The crystal structures generated during the current study are available in the Protein Data Bank (PDB) under the accession numbers 7UXX (apo CTD) and 7UXZ (CTD-CA), and through the links: https://www.rcsb.org/structure/7UXX and https://www.rcsb.org/structure/7UXZ, respectively.
