# Supplementary material for: Discovery and structural characterization of chicoric acid as a SARS-CoV-2 nucleocapsid protein ligand and RNA binding disruptor
Source: Sci Rep. 2022 Nov 2;12:18500. doi: 10.1038/s41598-022-22576-4 (PMC9628480; doi:10.1038/s41598-022-22576-4)
Supplement: Supplementary file 1 — Supplementary Information. [file 41598_2022_22576_MOESM1_ESM.docx]

**Supporting Information**

**Discovery and structural characterization of chicoric acid as a SARS-CoV-2 nucleocapsid protein ligand and RNA binding disruptor**

Gustavo Fernando Mercaldi^1a^, Eduardo Henrique Salviano Bezerra^1a^, Fernanda Aparecida Heleno Batista^1a^, Celisa Caldana Costa Tonoli^1^, Adriana Santos Soprano^1^, Jacqueline Farinha Shimizu^1^, Alice Nagai^1^, Jaqueline Cristina da Silva^1^, Helder Veras Ribeiro Filho^1^, Jéssica do Nascimento Faria^1^, Marcos Guilherme da Cunha^1^, Ana Carolina Mattos Zeri^2^, Andrey Ziem Nascimento^2^, José Luiz Proenca-Modena^3,4^, Marcio Chaim Bajgelman^1^, Silvana Aparecida Rocco^1^, Paulo Sérgio Lopes de Oliveira^1^, Artur Torres Cordeiro^1^, Marjorie Bruder^1^, Rafael Elias Marques^1^, Mauricio Luis Sforça^1^, Kleber Gomes Franchini^1^, Celso Eduardo Benedetti^1^, Ana Carolina Migliorini Figueira^1*^, Daniela Barretto Barbosa Trivella^1*^

^1^Brazilian Biosciences National Laboratory (LNBio), Brazilian Centre for Research in Energy and Materials (CNPEM), 13083-100, Campinas, SP, Brazil

^2^Brazilian Synchrotron Light Source (LNLS), Brazilian Centre for Research in Energy and Materials (CNPEM), 13083-100, Campinas, SP, Brazil

^3^Laboratory of Emerging Viruses (LEVE), Department of Genetics, Evolution, Microbiology and Immunology, Institute of Biology, University of Campinas (UNICAMP), Campinas, SP, Brazil

^4^Hub of Global Health (HGH), University of Campinas (UNICAMP), Campinas, Brazil

^a^ authors contributed equally to this work

*Corresponding authors:

Daniela B. B. Trivella

phone: +55 19 3517-5055

E-mail: [daniela.trivella@lnbio.cnpem.br](mailto:daniela.trivella@lnbio.cnpem.br)

Ana Carolina M. Figueira

phone: +55 19 3512-1100

E-mail: [ana.figueira@lnbio.cnpem.br](mailto:ana.figueira@lnbio.cnpem.br)

**TABLES**

## Table S1. X-ray data collection and refinement statistics.

| **Parameter/ structure** | **Apo CTD** | **CTD Chicoric acid** |
| --- | --- | --- |
| **Data colection** |  |  |
| **Source** | Manacá, Sirius | Manacá, Sirius |
| **Wavelength (****Å)** | 1.32 | 0.97 |
| **Resolution range (Å)** | 41.3 - 1.85 (1.916 - 1.85) | 43.98 - 1.73 (1.79 - 1.73) |
| **Space group** | *P* 2_1_ 2_1_ 2_1_ | *P* 2_1_ 2_1_ 2_1_ |
| **a b c (Å) – α β γ (^o^)** | 43.61 119.75 128.56 - 90 90 90 | 43.66 120.65 128.50 - 90 90 90 |
| **Total reflections** | 116966 (11518) | 756128 (70666) |
| **Unique reflections** | 58499 (5761) | 71415 (6959) |
| **Multiplicity** | 2.0 (2.0) | 10.6 (10.2) |
| **Completeness (%)** | 99.93 (99.97) | 99.86 (98.86) |
| **Mean I/sigma(I)** | 9.33 (2.66) | 9.46 (1.28) |
| **Wilson B-factor** | 16.21 | 18.25 |
| **R-merge** | 0.053 (0.245) | 0.403 (2.741) |
| **R-meas** | 0.075 (0.347) | 0.423 (2.885) |
| **R-pim** | 0.053 (0.245) | 0.127 (0.891) |
| **CC_1/2_** | 0.997 (0.887) | 0.995 (0.509) |
| **Reflections in refinement** | 58463 (5759) | 71415 (6959) |
| **Reflections for R-free** | 2914 (345) | 3571 (348) |
| **Refinement** |  |  |
| **R_work_ (%)** | 0.172 (0.199) | 0.174 (0.315) |
| **R_free_ (%)** | 0.203 (0.240) | 0.215 (0.324) |
| **CC(work)** | 0.963 (0.933) | 0.964 (0.716) |
| **CC(free)** | 0.947 (0.901) | 0.941 (0.638) |
| **RMS bonds(Å)** | 0.006 | 0.014 |
| **RMS angles(^o^)** | 1.40 | 1.83 |
| **Ramachandran statistics** |  |  |
| **Favored (%)** | 98.29 | 98.44 |
| **Allowed (%)** | 1.71 | 1.56 |
| **Outliers (%)** | 0.00 | 0.00 |
| **Rotamer outliers (%)** | 0.00 | 3.39 |
| **Clashscore** | 7.80 | 5.29 |
| **Average B-factor (Å^2^)** | 20.25 | 22.08 |
| **macromolecules** | 17.96 | 20.31 |
| **ligands** | 23.80 | 50.57 |
| **solvent** | 31.81 | 31.82 |

Statistics for the highest-resolution shell are shown in parentheses.

**FIGURES**

**Figure S1. Validation of a screening assay for the SARS-CoV-2 N protein.** A) FP assays showing that the binding affinity of the RNA1 probe for the N protein is not affected by 2% DMSO and 0.01% triton X-100, two additives absent in the binding buffer (50 mM Sodium Phosphate, 0.1 M NaCl, pH7.6) but present in the buffer used in the HTS trials. B) Scatter plot showing the mP values for positive and negative controls obtained for each HTS assay plate. The mean and standard deviation values for positive and negative controls were 220.2 ± 5.1 and 51.1 ± 3.2, respectively.

**Figure S2. The binding affinity of RNA1 to the N protein is reduced in the presence of CI, PG and CA and the phenolic acids CI and CA are not protein aggregators.** A) FP assay showing that when the N protein (concentration ranging from 0.61 nM up to 5000 nM), in the presence of CI, CA or PG at 1:2 ratio (protein:compound), is further titrated against RNA1, it displayed a reduced binding affinity for RNA1, as indicated by the corresponding KD values. Error bars represent standard deviation of the means of three independent experiments. B) DLS measurements of N protein samples incubated with two-fold molar excess of each compound (CI, CA or PG at 20 µM final concentration), showing that the phenolic acids CI and CA do not significantly change the Rh or the oligomeric state of the N protein. The phenolic aldehyde, PG, on the other hand, appears to cause N protein aggregation, as indicated by the variation in the Rh values obtained. Plotted values are the means of three independent measurements with error bars denoting standard deviations.

**Figure S3. The N protein SR linker is required for RNA1 binding**. A) FP assay showing that neither the NTD nor the CTD alone interacts with RNA1. RNA1 binding is observed with the NTD-L-CTD protein, where the NTD and CTD are joined by the SR linker. B) 1H-15N-HSQC experiments of labeled 15N-CTD before (black signals) and after (red signals) the addition of RNA1 (protein:RNA1 at 1:1 ratio), showing no significant changes in the amino acid chemical shifts between treatments.​

**Figure S4. Cell viability assays in the presence of CA.** Calu-3 and Vero CCL81 cells were treated with CA at 25 µM or 100 µM for 48 h. Cell viability was evaluated using the MTT assay. DMSO at 0.2% final concentration was used as vehicle control. Results are expressed as individual values (n=16) and error bars represent mean +/- standard deviation.

**A**

**B**

**Figure S5. Sequence and structural alignment of SARS-CoV-2, SARS-CoV and MERS N proteins CTDs show conservation of the CA-binding site among these pathogenic human coronaviruses.** A) Multiple sequence alignment with CLUSTAL O (1.2.4). B) Superposition of the N protein CTDs crystal structures for SARS-CoV-2 (PDB ID 7UXZ, grey), SARS-CoV (PDB entry 2GIB, cyan) and MERS (PDB entry 6G13, magenta). The residues involved in CA binding are shown as sticks and labelled.

**A**

**B**
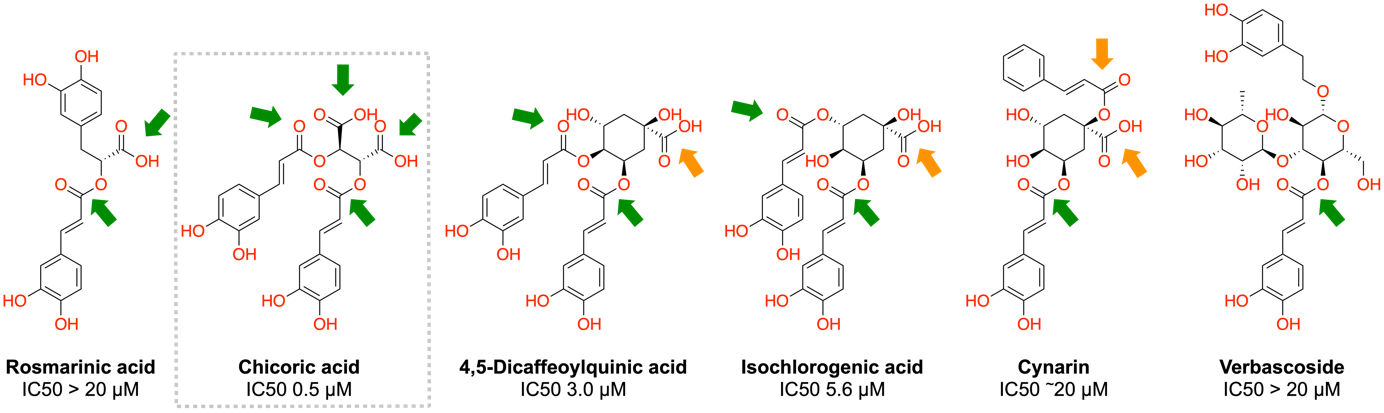


**Figure S6. Initial structure-activity relationship (SAR) analysis of 16 compounds containing caffeoyl substructures assessed in our screening efforts against SARS-CoV-2 N protein suggests that both dicaffeoyl carbonyls and symmetric carboxylates are key pharmacophores for the CA-N protein biding site.** A) Sixteen compounds assessed in our screening efforts and containing caffeoyl substructures, those bearing two caffeoyl esters clearly stand out with over 50% N protein-RNA1 disruption at 20 µM. B) In CA, the most potent derivative (IC_50_ = 0.5 µM), the dicaffeoyl units are bound to tartaric acid, whose carboxylate moieties’ contribution for protein binding have been established in the present work. The other three dicaffeoyl derivatives are in fact isomers of dicaffeoylquinic acid, being 4,5-dicaffeoylquinic acid, 3,5-dicaffeoylquinic acid (isochlorogenic acid A) and 1,5-dicaffeoylquinic acid (cynarin). Assuming that they bind to the same site as CA, one can see that the caffeoyl esters could occupy similar positions, especially for 4,5-dicaffeoylquinic acid and isochlorogenic acid A. Their single carboxylate moiety at C1 would be further way in comparison to CA, but still able to engage in ionic interactions with positively charged residues, but clearly, this modification causes a ~10-fold loss in IC_50_ values. Looking at cynarin or 1,5-dicaffeoylquinic acid, one could propose that in order to accommodate the caffeoyl esters similarly to its congeners and CA, the carboxylate moiety would be moved from the arginine-rich region, yielding a much weaker binding affinity. Or one could suggest that in order to achieve ionic interactions between the carboxylate and arginine residues, the caffeoyl ester moieties would lose contact with the protein, likewise yielding low binding affinity. This observation can be extended to the other caffeoyl derivatives, such as rosmarinic acid and verbascoside which showed low N protein/RNA1 disruption, and which only contain one or two of the 4 key elements found in CA. Indeed, the two carboxylate and two caffeoyl ester moieties are important for binding to the N protein and to induce conformational changes that affect its RNA binding function.
